# Identification of a pyroptosis‐based model for predicting clinical outcomes from immunotherapy in patients with metastatic melanoma

**DOI:** 10.1002/cam4.5178

**Published:** 2022-09-23

**Authors:** Guanghao Wu, Biying Chen, Junjie Jiang, Yiran Chen, Yanyan Chen, Haiyong Wang

**Affiliations:** ^1^ School of Clinical Medicine Hangzhou Normal University Medical College Hangzhou China; ^2^ Department of Surgical Oncology The First Affiliated Hospital, Zhejiang University School of Medicine Hangzhou China; ^3^ Department of Gastroenterology Affiliated Hangzhou First People's Hospital, Zhejiang University School of Medicine Hangzhou China

**Keywords:** durable clinical benefit, immunotherapy, melanoma, predictive model, pyroptosis

## Abstract

Immunotherapy has greatly improved outcomes for patients with advanced melanoma, but good predictive biomarkers remain lacking in clinical practice. Although increasing evidence has revealed a vital role of pyroptosis in the tumor microenvironment (TME), it remains unclear for pyroptosis as a predictive biomarker for immunotherapy in melanoma. RNA sequencing data and annotated clinical information of melanoma patients were obtained from four published immunotherapy datasets. LASSO regression analysis was conducted to develop a pyroptosis‐based model for quantifying a pyroptosis score in each tumor. Based on four clinical cohorts, we evaluated the predictive capability of the model using multiple immunotherapeutic outcomes, including clinical benefits, overall survival (OS), and progression‐free survival (PFS). Furthermore, we depicted the distinctive TME features associated with pyroptosis. Compared with the group with low pyroptosis scores, the group with high pyroptosis scores consistently achieved better durable clinical benefits in four independent cohorts and the meta‐cohort. ROC analysis validated that the pyroptosis‐based model was a reliable biomarker for predicting clinical benefits from immunotherapy in melanoma. Survival analyses showed that the group with high pyroptosis scores harbored more favorable OS and PFS than those with low pyroptosis scores. Molecular analysis revealed that tumors with high pyroptosis scores displayed a typical immune‐inflamed phenotype in TME, including enrichment of immunostimulatory pathways, increased level of tumor‐infiltrating lymphocytes, upregulation of immune effectors, and activation of the antitumor immune response. Our findings suggested that the pyroptosis‐related model associated with multiple immune‐inflamed characteristics might be a reliable tool for predicting clinical benefit and survival outcomes from immunotherapy in melanoma.

## Graphical abstract

This study identified the pyroptosis‐related model associated with multiple immune‐inflamed characteristics as a reliable tool for predicting clinical benefit and survival outcomes from immunotherapy in melanoma. Preliminary data from four published cohorts consistently suggested better treatment outcomes from immunotherapy in melanoma with high pyroptosis scores, which was characterized as the immune‐inflamed phenotype in TME.

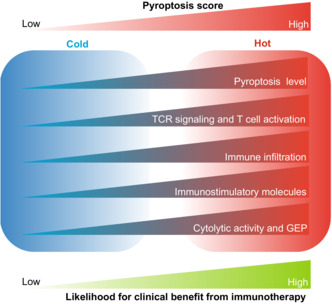

## 1 INTRODUCTION

Melanoma is the most malignant cutaneous neoplasm which arises from melanocytes.^1^ According to GLOBOCAN 2020, cutaneous melanoma was responsible for approximately 354,635 new cases and 57,043 deaths, accounting for 1.7% of tumor incidence and 0.6% of tumor mortality.^2^ Furthermore, it causes the majority (75%) of deaths associated with cutaneous neoplasm due to high invasion and early metastasis.^3^, ^4^ In the past decade, the advent of immunotherapy has dramatically shifted the treatment pattern in melanoma.^5^ The development of multimodal immunotherapy has brought several promising treatment options for metastatic or unresectable advanced melanoma, such as anti‐PD‐1/PD‐L1 inhibitors, engineered TCR therapy, adoptive T cell therapy, vaccines, and combination strategies. However, due to the nonnegligible heterogeneity among different populations,^7^ only a limited number of melanoma patients could achieve durable clinical benefits from immunotherapy in clinical practice.^8^ Therefore, developing a predictive biomarker for screening patients who might respond to immunotherapy is of great clinical significance.

Pyroptosis is a novel form of programmed necrotic cell death mediated by the gasdermin (GSDM) protein family,^9^, ^10^ mainly characterized by inflammasome activation, caspase activation, and pore formation in the cell membrane.^11^ Pyroptosis can cause permeable cell swelling and rupture accompanied by the production of inflammatory cytokines and chemokines, resulting in a potent inflammatory response.^12^ Initially, pyroptosis was considered an inflammatory form of the innate immune response to defend the host infection from pathogens.^13^ Recently, pyroptosis has been considered a promising opportunity to reduce immunosuppression and enhance the antitumor immune response.^14^, ^15^, ^16^ Single‐cell RNA sequencing analysis suggested that inducing pyroptosis could promote the recruitment of tumor‐infiltrating immunostimulatory cells and inhibit the activation of immunosuppressive cells in breast cancer.^17^ Furthermore, a molecular classification study found that subtypes with high pyroptosis‐related gene expression belonged to the immunological “hot” phenotype with activated immune response in bladder cancer.^18^ Bioinformatic analyses also identified a series of pyroptosis‐based signatures associated with immune status and prognosis in melanoma.^19^, ^20^ However, the relevance of pyroptosis with the efficacy of tumor immunotherapy remains unclear in melanoma.

This study developed a pyroptosis‐based model to predict clinical benefits from immunotherapy in melanoma using four published immunotherapy cohorts.^21^, ^22^, ^23^, ^24^ In addition, we explored the prognostic value of the pyroptosis‐based model in melanoma patients with or without immunotherapy. Furthermore, we depicted the unique tumor microenvironment (TME) characteristics associated with the pyroptosis‐based model to explain the predictive value for immunotherapy.

## 2 MATERIALS AND METHODS

### 2.1 Datasets collection

We performed a literature search in the Medline, Web of Science, and EMBASE databases to retrieve the public available immunotherapy cohorts. The keywords included melanoma, immunotherapy, and RNA sequencing. Studies were included when met the following criteria: (1) tumor samples were pathologically confirmed as melanoma; (2) patients received at least one kind of immunotherapy; (3) original data on response, clinical benefit, or survival outcomes were provided; (4) studies were published in English; (5) RNA‐seq data were available. The exclusion criteria were as follows: (1) studies without detailed patient information; (2) duplicated studies; (3) review, letter, editorial, comment, abstract, and case report. As a result, this study enrolled four eligible cohorts with RNA‐seq and related clinical data, including the Gide,^21^ Lauss,^23^ Liu,^22^ and Nathanson^24^ cohorts. The Gide cohort contained 73 melanoma patients receiving anti‐PD‐1 monotherapy or the combination between anti‐PD‐1 and anti‐CTLA‐4 therapy. The Lauss cohort consisted of 25 melanoma patients receiving adoptive T cell therapy. The Liu cohort included 119 melanoma patients receiving anti‐PD‐1 monotherapy. The Nathanson cohort contained 21 melanoma patients receiving anti‐CTLA‐4 monotherapy. In addition, the clinicopathological features, including age, gender, M stage, primary tumor site, therapy type, therapeutic response, clinical benefit, and survival outcomes, were recorded in Table 1. This study aimed to develop an effective biomarker for immunotherapy in melanoma. The Gide cohort was selected as the training cohort, and the others were chosen as validation cohorts. Besides, the RNA sequencing with annotated clinical data of the TCGA‐SKCM cohort was downloaded from the cBioPortal database. After removing the cases with incomplete RNA sequencing and clinical data, a total of 402 melanoma patients were enrolled in the TCGA‐SKCM cohort.

**TABLE 1:** Baseline clinical characteristics of patients with melanoma in four immunotherapy cohorts

Characteristic	Gide cohort (*N* = 73)	Lauss cohort (*N* = 25)	Liu cohort (*N* = 119)	Nathanson cohort (*N* = 21)
Age ‐ yrs				
Median	62	NA	NA	58
Range	24–90	NA	NA	33–90
Gender ‐ no. (%)				
Female	47 (64.4)	NA	49 (41.2)	11 (52.4)
Male	26 (35.6)	NA	70 (58.8)	10 (47.6)
Tumor site ‐ no. (%)				
Cutaneous	NA	17 (68.0)	87 (73.1)	16 (76.2)
Mucosal	NA	2 (8.0)	6 (5.0)	0 (0.0)
Acral	NA	0 (0.0)	7 (5.9)	3 (14.3)
Occult	NA	6 (24.0)	19 (16.0)	2 (9.5)
Stage ‐ no. (%)				
M0	NA	0 (0.0)	10 (8.4)	0 (0.0)
M1a	NA	1 (4.0)	7 (5.9)	0 (0.0)
M1b	NA	3 (12.0)	14 (11.8)	4 (19.0)
M1c	NA	21 (84.0)	88 (73.9)	17 (81.0)
Immunotherapy ‐ no. (%)				
Anti‐PD‐1	41 (56.2)	0 (0.0)	119 (100.0)	0 (0.0)
Anti‐CTLA‐4	0 (0.0)	0 (0.0)	0 (0.0)	21 (100.0)
Anti‐PD‐1+ anti‐CTLA‐4	32 (43.8)	0 (0.0)	0 (0.0)	0 (0.0)
Adoptive T cell	0 (0.0)	25 (100.0)	0 (0.0)	0 (0.0)
Best objective response ‐ no. (%)				
CR	14 (19.2)	5 (20.0)	16 (13.4)	NA
PR	26 (35.6)	5 (20.0)	31 (26.1)	NA
SD	11 (15.1)	10 (40.0)	16 (13.4)	NA
PD	22 (30.1)	5 (20.0)	56 (47.1)	NA
Durable clinical benefit ‐ no. (%)				
Yes	46 (63.0)	10 (40.0)	61 (51.3)	8 (38.1)
No	27 (37.0)	15 (60.0)	58 (48.7)	13 (61.9)
Overall survival ‐ mos				
Median	20.8	22.8	18.1	24.5
Range	0.7–53.4	3.2–92.9	1.3–56.4	4.8–94.6
Progression‐free survival ‐ mos				
Median	12.9	3.8	5.1	NA
Range	0.4–53.4	1.9–67.1	0.4–56.0	NA

### 2.2 Construction of the pyroptosis‐based model for predicting response to immunotherapy

First, the geneset containing 33 pyroptosis‐related genes was collected from previous studies,^25^, ^26^, ^27^ as shown in Table S1. The gene expression matrix with annotated clinical information was extracted from the Gide cohort. Second, LASSO regression analysis was conducted by the R package “glmnet” to identify the pyroptosis‐related genes closely associated with clinical benefits from immunotherapy. The gene number was determined according to the ideal penalty (Lambda). Third, the gene coefficient (coef) was obtained from the LASSO regression model. Subsequently, a pyroptosis‐based model, named pyroptosis score, was constructed according to the combination between the coefficient (coef) and gene expression level as follows: pyroptosis score = ∑ (coef _i_ × gene _i_). Following that, the pyroptosis score of each tumor sample in the Gide, Lauss, Liu, and Nathanson cohorts was determined using this formula. The distribution of the pyroptosis score in each cohort was visualized using the R package “waterfalls”. In addition, four machine learning methods, including random forests (RF), support vector machines (SVM), artificial neural networks (ANN), and K‐nearest neighbor (KNN), were performed by the R package “Caret” to build models for predicting clinical benefits from immunotherapy in melanoma.

### 2.3 ROC analysis and survival analysis

The predictive efficiency for clinical benefits from immunotherapy was tested using receiver operating characteristic (ROC) analyses in the Gide, Lauss, Liu, and Nathanson cohorts. The area under the ROC curve (AUC) was determined using the R package “pROC”. In addition, Kaplan–Meier analysis was conducted to evaluate the prognostic value of the pyroptosis model in melanoma patients with immunotherapy. The median value of the pyroptosis score was adopted as the grouping threshold.

### 2.4 Logistic regression analysis, Cox regression analysis, and meta‐analysis

The Odds ratios (OR) and 95% confidence interval (CI) were determined using univariate logistic regression analysis to estimate the difference in DCB between the groups with different pyroptosis scores. The hazard ratio (HR) and 95% CI were determined using univariate Cox regression analysis to assess the association of the pyroptosis score with OS and PFS. Based on these results, a meta‐analysis was conducted using the R package “meta” to evaluate the combined effect and the interstudy heterogeneity. The interstudy heterogeneity was defined according to the value of *I* ^2^ as follows: low (*I* ^2^ < 25%), moderate (*I* ^2^ = 25–75%), or high (*I* ^2^ > 75%).^28^ A fixed‐effect model was applied in the meta‐analysis when *I* ^2^ was less than 50%. Otherwise, a random‐effect model was applied in the meta‐analysis.

### 2.5 Function annotation and pathway enrichment analysis

First, considering the limited number of samples in a single cohort and the low heterogeneity among the clinical cohorts, we merged the Gide, Lauss, Liu, and Nathanson cohorts as an overall cohort for molecular analyses. The batch effect from different cohorts was corrected using the R package “sva”. Then, the differentially expressed genes (DEGs) between the groups with different pyroptosis scores were screened using the R package “limma”. |Fold change (FC)| > 1.5 with the adjusted *p* < 0.05 was adopted as the thresholds. Based on these DEGs, Gene Ontology (GO), Kyoto Gene and Genomic Encyclopedia (KEGG), and gene set enrichment analysis (GSEA) analyses were performed using the R packages “GO.db”, “org.Hs.eg.db”, “topGO”, “DOSE”, “GSEABase”, “clusterProfiler”, and “stringr”. For GSEA analysis, the REACTOME genesets were downloaded from the Molecular Signatures Database (MsigDB). The number of random sample permutations was adopted as 1000.

### 2.6 Evaluation of tumor‐infiltrating immune cells, TME scores, immune effectors, and transcriptional biomarkers for immunotherapy

CIBERSORT was a deconvolution algorithm widely applied to estimate tumor‐infiltrating immune cells, which output the relative infiltration level of immune cells. In this study, we also evaluated the infiltration level of 22 immune cell types in each tumor sample using the CIBERSORT algorithm. The R package “ESTIMATE” was applied to determine the TME scores, including ESTIMATE score, stromal score, and immune score, which reflected tumor purity, stromal content, and immune cell infiltration level. In addition, immune‐related genes were obtained from published literature,^29^, ^30^, ^31^, ^32^, ^33^, ^34^, ^35^ including activated DC markers, antigen‐processing related genes, CD8^+^ T effectors, genes downstream of IFN‐γ, NK markers, cytolysis molecules, and immune checkpoints. Furthermore, we explored the correlation between the pyroptosis score and several established transcriptional biomarkers for immunotherapy, including cytolytic activity (CYT)^34^ and the T cell–inflamed gene expression profile (GEP).^32^ As previously reported, CYT was determined by the mean expression of GZMA and PRF. GEP was determined by the mean expression of a T cell‐inflamed gene expression profile, including CD276, CD8A, HLA‐E, CXCL9, CXCR6, CCL5, PDCD1LG2, HLA‐DQA1, HLA‐DRB1, PSMB10, CD27, IDO1, LAG3, NKG7, CMKLR1, CD274, STAT1, and TIGIT.

### 2.7 Statistical analysis

All data analyses were performed in SPSS 21.0 and R software 3.6.1. The correlation between the pyroptosis score and the pyroptosis level, the immune effectors, and the transcriptional biomarkers for immunotherapy (CYT and GEP) were determined using Spearman's correlation analysis. Mann–Whitney test was applied to compare the difference in immune cell proportions and TME scores between the low and high pyroptosis groups. The comparison of clinical benefit rate was dealt with chi‐squared (*χ* ^2^) test or Fisher's exact test. The log‐rank test was used to compare Kaplan–Meier curves. A *P* value <0.05 was defined as statistically significant.

## 3 RESULTS

### 3.1 Baseline characteristics of melanoma patients with immunotherapy

We collected the baseline characteristics of 238 melanoma patients with immunotherapy from four previous studies, including the Gide,^21^ Lauss,^23^ Liu,^22^ and Nathanson^24^ cohorts. The Gide cohort consisted of 73 melanoma patients. 56.2% (41/73) received anti‐PD‐1 monotherapy, and 43.8% (32/73) received anti‐PD‐1 combined with anti‐CTLA‐4 therapy. Based on the RECIST v.1.1 criteria, the best objective response to immunotherapy included 19.2% (14/73) with CR, 35.6% (26/73) with PR, 15.1% (11/73) with SD, and 30.1% (22/73) with PD. Regarding the clinical benefit from immunotherapy, 63.0% (46/73) were with DCB, whereas 37.0% (27/73) were with NDB. The median value of OS and PFS was 20.8 months and 12.9 months, respectively. The Lauss cohort contained 25 melanoma patients who received adoptive T cell therapy. Most of them (84.0%) were at the M1c stage. The best objective response included 20.0% (5/25) with CR, 20.0% (5/25) with PR, 40.0% (10/25) with SD, and 20.0% (5/25) with PD. The percentage of DCB was 40.0%. The median value of OS and PFS was 22.8 months and 3.8 months, respectively. The Liu cohort consisted of 119 melanoma patients who received anti‐PD‐1 monotherapy. The best objective response included 13.4% (16/119) with CR, 26.1% (31/119) with PR, 13.4% (16/119) with SD, and 47.1% (56/119) with PD. The percentage of DCB was 51.3%. The median value of OS and PFS was 18.1 months and 5.1 months, respectively. Nathanson cohort contained 21 melanoma patients who received anti‐CTLA‐4 monotherapy. Most of them (81.0%) were at the M1c stage. The percentage of DCB was 38.1%, and the median value of OS was 24.5 months. Nevertheless, the data on response to immunotherapy and PFS were unavailable in the Nathanson cohort.

### 3.2 Construction of a pyroptosis‐based model for predicting DCB from immunotherapy in melanoma

**FIGURE 1:**
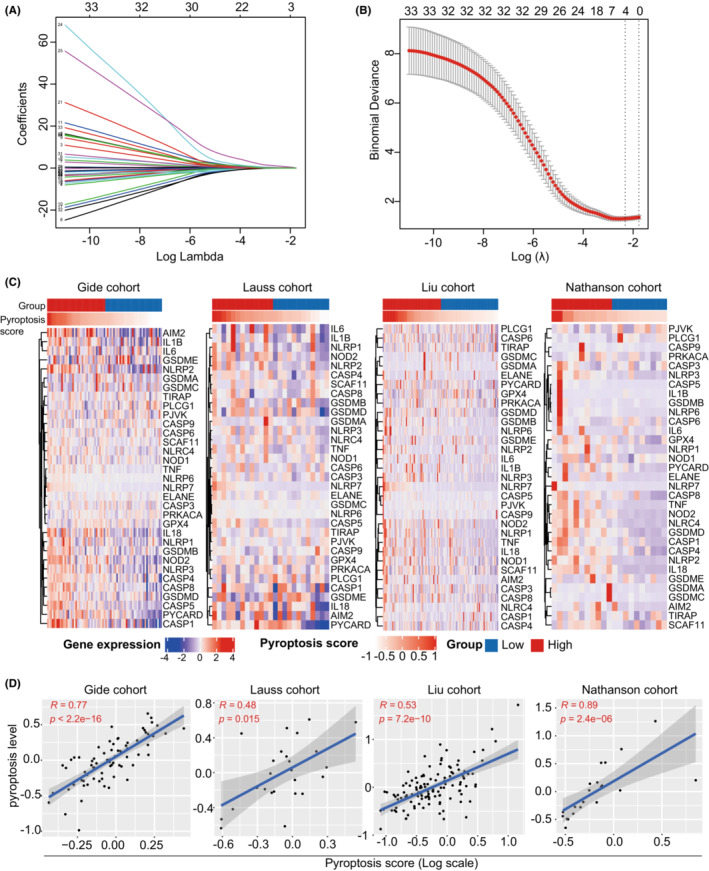
Development of the pyroptosis‐based model for predicting response to immunotherapy in melanoma. (A) LASSO regression analysis showed that the parameters decreased to zero when the penalty (Lambda) increased in the Gide cohort. (B) Four candidate variables (Lambda) were enrolled in the LASSO model, including CASP5, NLRP6, NLRP7, and PYCARD. A pyroptosis‐based model, named pyroptosis score, was constructed by combining gene expression with the corresponding coefficients based on these candidate variables. (C) Heatmap showing the pyroptosis‐related gene expression of melanoma samples in the Gide, Lauss, Liu, and Nathanson cohorts. This study selected the Gide cohort as the training cohort and the others as validation cohorts. The median value of the pyroptosis score was adopted as the threshold for dividing patients into two groups. Each row and column represent genes and tumor samples, respectively. The columns are ranked according to the pyroptosis score. (D) Correlation analysis between pyroptosis score and the pyroptosis level in the Gide, Lauss, Liu, and Nathanson cohorts. The pyroptosis level was determined by the mean expression of the pyroptosis‐related genes in tumor samples

The gene expression matrix was matched with the geneset of 33 pyroptosis‐related genes and extracted from the Gide cohort. Then, LASSO regression analysis was performed to screen candidate genes significantly associated with DCB from immunotherapy. As a result, four ideal genes were enrolled in the predictive model (Figure 1A,B). Based on the gene expression level with variable coefficient, a pyroptosis‐related model was developed according to the following formula: pyroptosis score = (0.139 × CASP5) + (0.240 × NLRP6) + (1.388 × NLRP7) + (0.112 × PYCARD). According to the predictive model, each tumor sample in four immunotherapy cohorts was assigned with a pyroptosis score. Following that, we investigate the association between pyroptosis score and 33 pyroptosis‐related genes in the immunotherapy cohorts. It was found that tumor samples with higher pyroptosis scores harbored more abundant expression levels of pyroptosis‐related genes in the Gide, Lauss, Liu, and Nathanson cohorts (Figure 1C). Subsequently, we quantified the pyroptosis level in each tumor sample using the mean expression level of 33 pyroptosis‐related genes. Correlation analysis showed that the pyroptosis scores were all significantly correlated with the pyroptosis level in the Gide (*R* = 0.77, *p* < 0.001), Lauss (*R* = 0.48, *p* < 0.001), Liu (*R* = 0.53, *p* < 0.001), and Nathanson (*R* = 0.89 *p* < 0.001) cohorts (Figure 1D).

### 3.3 Prediction of durable clinical benefit from immunotherapy by the pyroptosis‐based model

**FIGURE 2:**
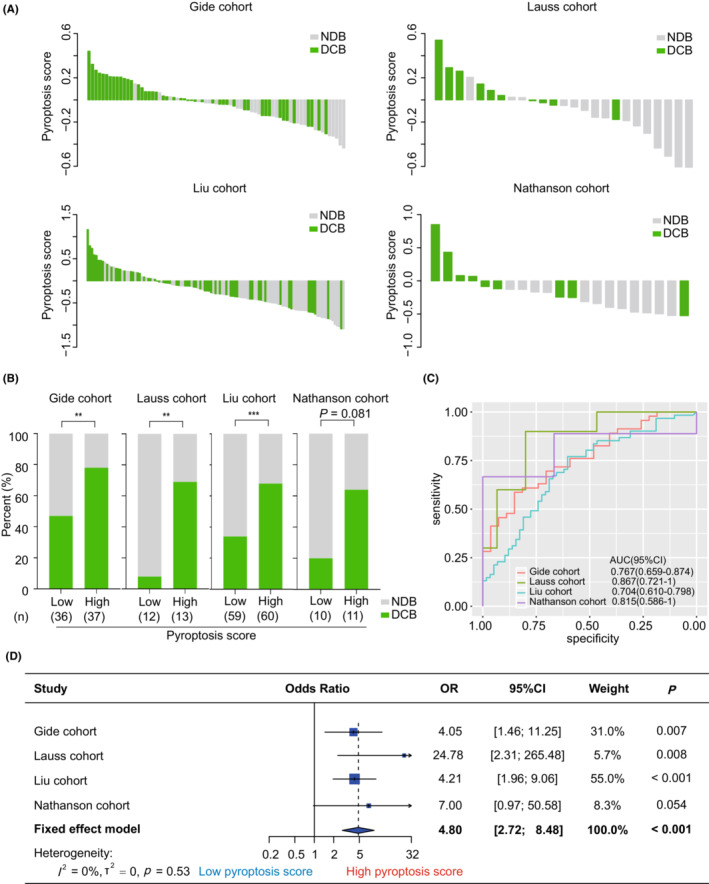
The association between the pyroptosis score and clinical benefit from immunotherapy in melanoma. (A) Waterfall plot showing the distribution of the pyroptosis score of patients with melanoma in the Gide, Lauss, Liu, and Nathanson cohorts. Green indicates durable clinical benefit, and gray indicates no durable clinical benefit. The definition of clinical benefit from immunotherapy referred to the RECIST v.1.1 criteria. (B) Comparison of the percentage of patients who got durable clinical benefit from immunotherapy between the groups with low and high pyroptosis scores in the Gide, Lauss, Liu, and Nathanson cohorts. (C) ROC curve showing the prediction of durable clinical benefit by pyroptosis score in the Gide, Lauss, Liu, and Nathanson cohorts. (D) Forest plot displaying the meta‐analysis for predicting durable clinical benefit from immunotherapy by pyroptosis score. The value of OR with corresponding 95% CI was determined using univariate logistic regression in the Gide, Lauss, Liu, and Nathanson cohorts. AUC, area under curve; CI, confidence interval; DCB, durable clinical benefit; NDB, no durable clinical benefit; OR, odds ratio; ^***^ *p* < 0.001, ^**^ *p* < 0.01

The pyroptosis‐based model was applied to predict the clinical benefits of immunotherapy in melanoma patients. First, we investigated the association of the pyroptosis score with DCB in the clinical cohorts. As shown in Figure 2A, a decreasing trend in the DCB proportion was observed along with the decrease in pyroptosis score in each cohort. We then compared the proportion of DCB between the groups with different pyroptosis scores. Expectedly, the group with high scores harbored a higher rate of DCB than the group with low scores in the Gide (78.4% vs 47.2%, *p* = 0.006, Figure 2B), Lauss (69.2% vs 8.3%, *p* = 0.004, Figure 2B), and Liu (68.3% vs 33.9%, *p* < 0.001, Figure 2B) cohorts. A similar trend was also observed in the Nathanson cohort, although it was not significant (63.6% vs 20.0%, *p* = 0.081, Figure 2B). Subsequently, ROC analysis suggested that the pyroptosis score was an effective and robust biomarker for predicting DCB from immunotherapy across various cohorts (Gide: AUC = 0.767, 95% CI = [0.659, 0.874]; Lauss: AUC = 0.867, 95% CI = [0.721, 1]; Liu: AUC = 0.704, 95% CI = [0.610, 0.798]; Nathanson: AUC = 0.815, 95% CI = [0.586, 1]; Figure 2C). Similar finding was observed in the combined cohort (AUC = 0.733, 95% CI = [0.670, 0.797], Figure S1). Furthermore, meta‐analysis validated that patients with high pyroptosis scores had a higher DCB rate than those with low pyroptosis scores (pooled OR = 4.80, 95% CI = [2.72, 8.48], *p* < 0.001, Figure 2D). The heterogeneity was mild among these cohorts (*I* ^2^ = 0%, tau^2^ = 0, *p* = 0.53, Figure 2D).

To compare the predictive value of different machine learning models, we further applied four machine learning methods, including random forests (RF), support vector machines (SVM), artificial neural networks (ANN), and K‐nearest neighbor (KNN), to build models for predicting clinical benefits from immunotherapy in melanoma. As shown in Figure S2, the AUC values of the RF, SVM, ANN, and KNN models were 1, 0.960, 0.856, and 0.792 in the training cohort (the Gide cohort), respectively. Nevertheless, the performance of these models in the validation cohorts was not as satisfactory as in the training cohort. Detailly, the AUC values of the RF model were 0.693, 0.545, and 0.721 in the Lauss, Liu, and Nathanson cohorts, respectively (Figure S2A). The AUC values of the SVM model were 0.733, 0.585, and 0.615 in the Lauss, Liu, and Nathanson cohorts, respectively (Figure S2B). The AUC values of the ANN model were 0.667, 0.553, and 0.500 in the Lauss, Liu, and Nathanson cohorts, respectively (Figure S2C). As for the KNN model, the AUC values were 0.673, 0.560, and 0.418 in the Lauss, Liu, and Nathanson cohorts, respectively (Figure S2D). However, compared with these models, the LASSO regression model used in this study harbored a more robust predictive performance (all AUC >0.7, Figure 2C).

### 3.4 Association of the pyroptosis‐based model with OS and PFS in the immunotherapy cohorts

**FIGURE 3:**
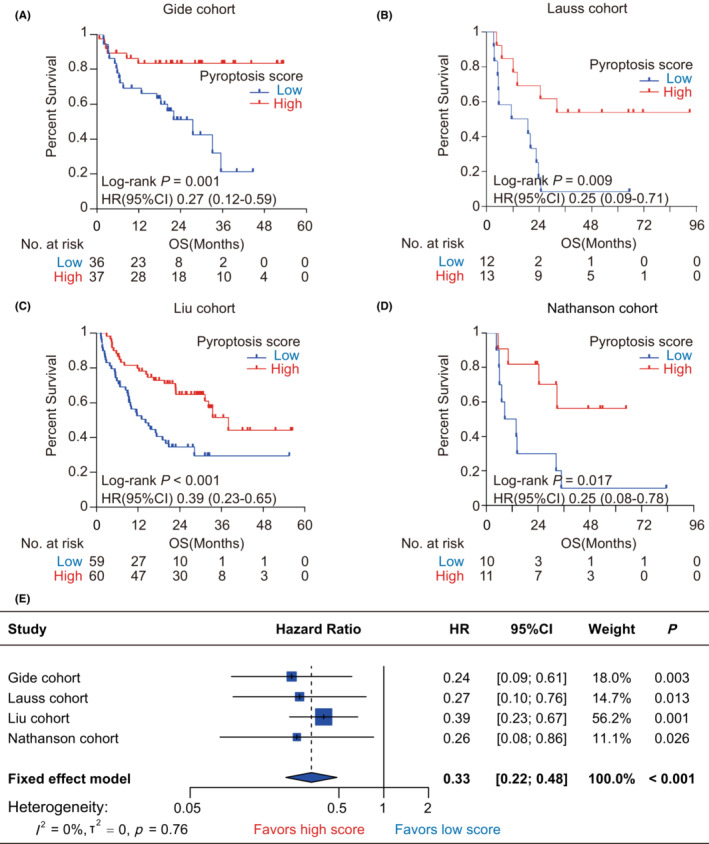
The association between pyroptosis score and overall survival in melanoma patients with immunotherapy. (A‐D) Kaplan–Meier analysis in the Gide (A), Lauss (B), Liu (C), and Nathanson (D) cohorts. (E) Forest plot displaying the meta‐analysis for the prediction of overall survival by pyroptosis score. The value of HR with corresponding 95% CI was determined using univariate Cox regression in the Gide, Lauss, Liu, and Nathanson cohorts. CI, confidence interval; HR, hazard ratio; OS, overall survival

**FIGURE 4:**
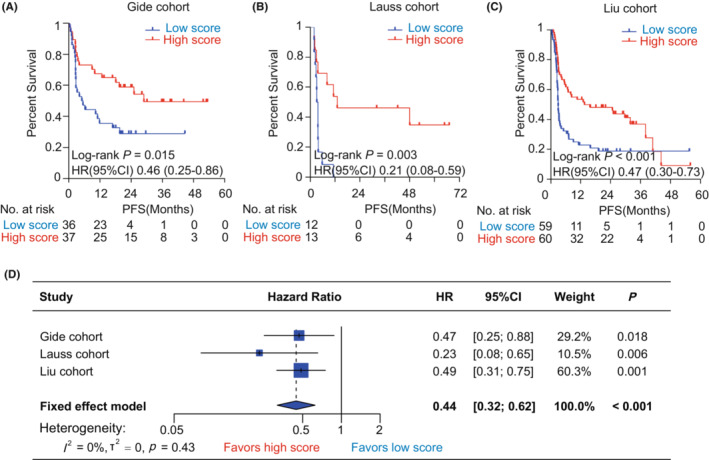
The association between pyroptosis score and progression‐free survival in melanoma patients with immunotherapy. (A‐C) Kaplan–Meier analysis in the Gide cohort (A), Lauss cohort (B), and Liu cohort (C). (D) Forest plot displaying the meta‐analysis for predicting progression‐free survival by pyroptosis score. The value of HR with corresponding 95% CI was determined using univariate Cox regression in the Gide, Lauss, Liu, and Nathanson cohorts. CI, confidence interval; HR, hazard ratio; PFS, progression‐free survival

We performed Kaplan–Meier analysis to evaluate the prognostic value of pyroptosis‐based model on OS and PFS in the immunotherapy cohorts. First, we observed that pyroptosis scores had a significantly protective value on OS in the immunotherapy cohorts (Gide: HR = 0.27, 95% CI = [0.12, 0.59], *p* = 0.001, Figure 3A; Lauss: HR = 0.25, 95% CI = [0.09, 0.71], *p* = 0.009, Figure 3B; Liu: HR = 0.39, 95% CI = [0.23, 0.65], *p* < 0.001, Figure 3C; Nathanson: HR = 0.25, 95% CI = [0.08, 0.78], *p* = 0.017, Figure 3D). Similarly, Cox regression analyses showed that patients with high pyroptosis scores consistently harbored a lower risk of OS than those with low pyroptosis scores across diverse cohorts (Gide: HR = 0.24, 95% CI = [0.09, 0.61], *p* = 0.003; Lauss: HR = 0.27, 95% CI = [0.10, 0.76], *p* = 0.013; Liu: HR = 0.39, 95% CI = [0.23, 0.67], *p* = 0.001; Nathanson: HR = 0.26, 95% CI = [0.08, 0.86], *p* = 0.026, Figure 3E). Furthermore, meta‐analysis validated that the pyroptosis score had a great and robust ability for distinguishing overall survival risk stratifications in melanoma patients receiving immunotherapy (pooled HR = 0.33, 95% CI = [0.22, 0.48], *p* < 0.001; *I* ^2^ = 0%, tau^2^ = 0, *p* = 0.76; Figure 3E). As for PFS, the pyroptosis‐based model also harbored a significant prognostic value. Kaplan–Meier analysis showed that patients with high pyroptosis scores all harbored a more favorable PFS than those with low pyroptosis scores in the Gide (HR = 0.46, 95% CI = [0.25, 0.86], *p* = 0.015, Figure 4A), Lauss (HR = 0.21, 95% CI = [0.08, 0.59], *p* = 0.003, Figure 4B), and Liu (HR = 0.47, 95% CI = [0.30, 0.73], *p* < 0.001, Figure 4C) cohorts. Meta‐analysis identified the pyroptosis score as a protective factor for PFS in melanoma patients receiving immunotherapy (HR = 0.44, 95% CI = [0.32, 0.62], *p* < 0.001, Figure 4D). Additionally, it remains unknown that the prognostic value of the pyroptosis‐based model for melanoma patients who did not receive immunotherapy. Therefore, we further explored the association of the pyroptosis score with OS and PFS in TCGA‐SKCM cohort. It was found that the differences of OS and PFS between the groups with low and high pyroptosis scores were both not significant in melanoma patients without any immunotherapy (Figure S3).

### 3.5 Predictive and prognostic value of the pyroptosis‐based model in patients with other cancer types receiving immunotherapy

To evaluate the applicability of the pyroptosis‐based model in other carcinomas, we collected two immunotherapy datasets from published cancer‐related literature, including metastatic gastric cancer (GC)^36^ and advanced clear cell renal cell carcinoma (ccRCC).^37^ The Kim cohort consisted of 45 GC patients who received pembrolizumab therapy.^36^ The Braun cohort contained 281 ccRCC patients treated with nivolumab.^37^ ROC analyses showed that the predictive accuracy of pyroptosis‐based model was not effective enough in the Kim and Braun cohorts (Kim: AUC = 0.548, 95% CI = [0.372, 0.724], Figure S4A; Braun: AUC = 0.504, 95% CI = [0.433, 0.575], Figure S4B). Furthermore, Kaplan–Meier analyses revealed that the differences in OS and PFS were both insignificant between the groups with low and high pyroptosis scores in the Braun cohort (OS: HR = 0.97, 95% CI = [0.73, 1.27], *p* = 0.815, Figure S4C; PFS: HR = 1.04, 95% CI = [0.81, 1.32], *p* = 0.772, Figure S4D). Taken together, we considered that the predictive value of the pyroptosis‐based model is specific for metastatic melanoma patients who received immunotherapy.

### 3.6 Function annotation and pathway enrichment of the pyroptosis‐based model

**FIGURE 5:**
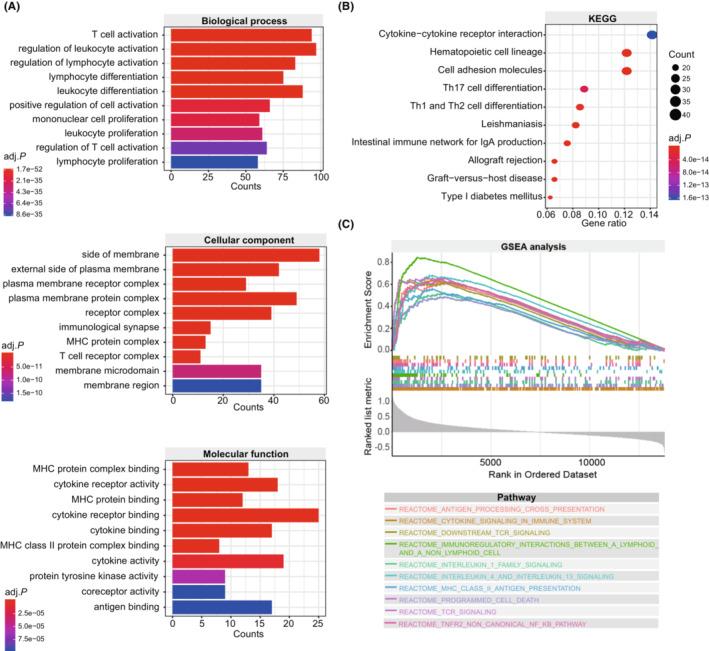
Function annotation and pathway enrichment of differentially expressed genes between the groups with low and high pyroptosis scores. (A) Histogram showing GO enrichment terms including BP, CC, and MF. (B) Bubble graph showing KEGG pathway enrichment. (C) GSEA analysis. The lower panel shows that the pyroptosis score decreases from left to right. BP, biological process; CC, cellular component; GO, Gene Ontology; GSEA, Gene set enrichment analysis; KEGG, Kyoto Encyclopedia of Genes and Genomes; MF, molecular function

The pyroptosis‐based model harbored a reliable and robust predictive value for melanoma patients treated with immunotherapy. Therefore, it is valuable to investigate the molecular basis underlying the model's predictive value. First, we screened 673 DEGs between tumor samples with different pyroptosis scores using the R package “limma”. Subsequently, we conducted GO function annotation and KEGG pathway enrichment analyses to investigate the biological functions of DEGs. Interestingly, DEGs were annotated with immune‐related biological functions (Figure 5A). At the biological process level, DEGs were mainly involved in the T cell activation, regulation of leukocyte activation, and regulation of lymphocyte activation. At the level of the cellular component, DEGs were mostly annotated with the side of the membrane, external side of plasma membrane, and plasma membrane receptor complex. At the molecular function level, DEGs presented a series of immune‐related functions, such as MHC protein complex binding, cytokine receptor activity, and MHC protein binding. KEGG pathway enrichment analysis showed that immune‐related pathways account for 70% of the top10 signaling pathways, including cytokine‐cytokine receptor interaction, hematopoietic cell lineage, Th17 cell differentiation, Th1 and Th2 cell differentiation, intestinal immune network for IgA production, allograft rejection, and graft‐versus‐host disease (Figure 5B). Furthermore, GSEA analysis showed that pyroptosis score was positively correlated with several immune‐related pathways, including antigen processing cross presentation, cytokine signaling in the immune system, downstream TCR signaling, immunoregulatory interactions between a lymphoid and a non‐lymphoid cell, interleukin‐1 family signaling, interleukin‐4 and interleukin‐13 signaling, MHC class II antigen presentation, programmed cell death, TCR signaling, and TNFR2 non‐canonical NF‐kβ pathway (All adjusted *p* < 0.05, Figure 5C).

### 3.7 Association between the pyroptosis‐based model and tumor immune microenvironment in melanoma

**FIGURE 6:**
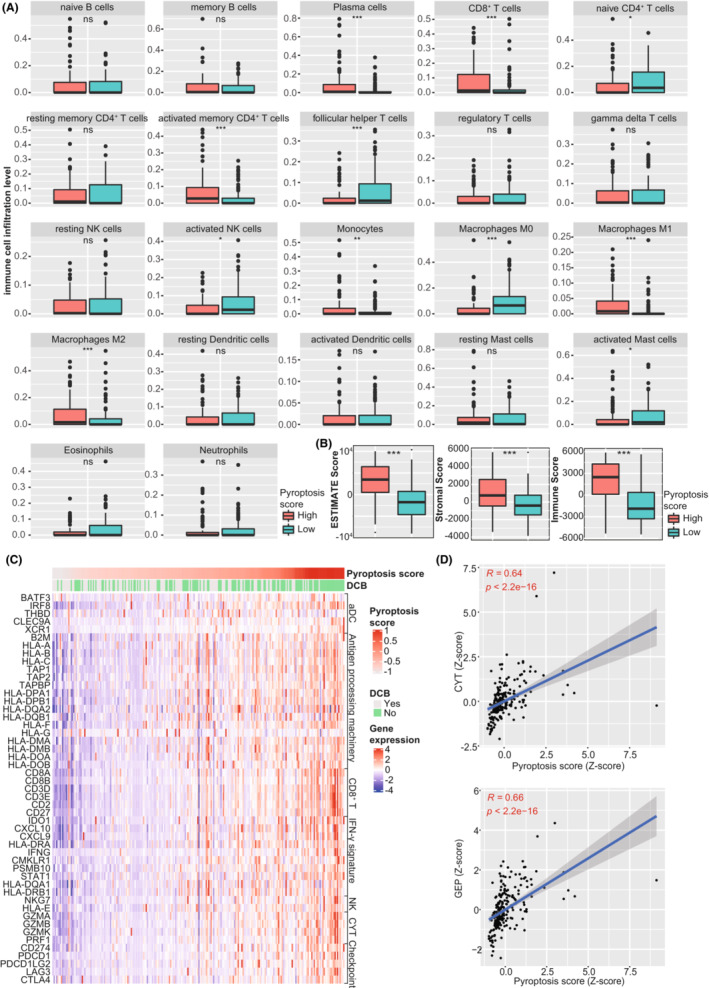
Association between pyroptosis score and tumor immune microenvironment in melanoma. (A) Comparison of the infiltration level of immune cells between the groups with low and high pyroptosis scores. The infiltration level of 22 immune cells was estimated using the CIBERSORT algorithm. (B) Comparison of three scores (ESTIMATE, stromal, and immune scores) between the groups with low and high pyroptosis scores. The ESTIMATE, stromal, and immune scores were determined using the ESTIMATE algorithm, which reflected the tumor purity, stromal content, and immune infiltration, respectively. (C) Correlation between pyroptosis score and immune effectors. The immune effectors included activated DC markers, antigen‐processing related genes, CD8^+^ T effectors, genes downstream of IFN‐γ, NK markers, cytolysis molecules, and immune checkpoints. (D) Correlation of pyroptosis score with CYT and GEP. aDC, activated DC cells; CYT, cytolytic activity; DCB, durable clinical benefit; GEP, T cell‐inflamed gene expression profile; NK, natural killer cells; *p < 0.05, **p < 0.01, ***p < 0.001; ns, not significant

Considering the significant association between DEGs and many immune‐related signaling pathways, we further explore the pyroptosis‐based model's role in melanoma's tumor immune microenvironment. First, we performed CIBERSORT algorithm to estimate the relative proportion of tumor‐infiltrating immune cells. Then we compared the abundance of 22 immune cell types between the groups with different pyroptosis scores. We observed that tumors with high pyroptosis scores harbored significantly elevated infiltration level of CD8^+^ T cells (*p* < 0.001), activated memory CD4^+^ T cells (*p* < 0.001), polarized Macrophages M1 (*p* < 0.001) and M2 (*p* < 0.001), and plasma cells (*p* < 0.001) than those with low pyroptosis scores (Figure 6A). On the contrary, tumors with low pyroptosis scores contained more resting immune cells, including naïve CD4^+^ T cells (*p* < 0.05) and macrophage M0 (*p* < 0.001). Second, the ESTIMATE algorithm was applied to evaluate TME scores in tumor samples. We observed that tumors with high pyroptosis scores had significantly higher ESTIMATE (*p* < 0.001), stromal (*p* < 0.001), and immune scores (*p* < 0.001) than those with low pyroptosis scores (Figure 6B), suggesting that tumors with high pyroptosis scores had more abundant TME composition, more stromal content, and elevated infiltration level of the immune cells. In addition, we found that tumors with high pyroptosis scores harbored more immune effectors, including the activated DC cell markers, antigen‐processing related genes, CD8^+^ T cell effectors, genes downstream of IFN‐γ, NK cell markers, and immune checkpoints (All *p* < 0.05, Figure 6C and Table S2). Besides, previous studies identified CYT and GEP as the predictive biomarkers at the transcriptional level to predict response to immunotherapy in various malignancies, which reflected T cell cytolytic activity and inflammation level in the tumor microenvironment.^32^ ^,^ ^34^ This study further investigated the correlation between the pyroptosis‐based model and these biomarkers. There was a significantly positive correlation of the pyroptosis score with CYT (*R* = 0.64, *p* < 0.001, Figure 6D) and GEP (*R* = 0.66, *p* < 0.001, Figure 6D).

**FIGURE 7:**
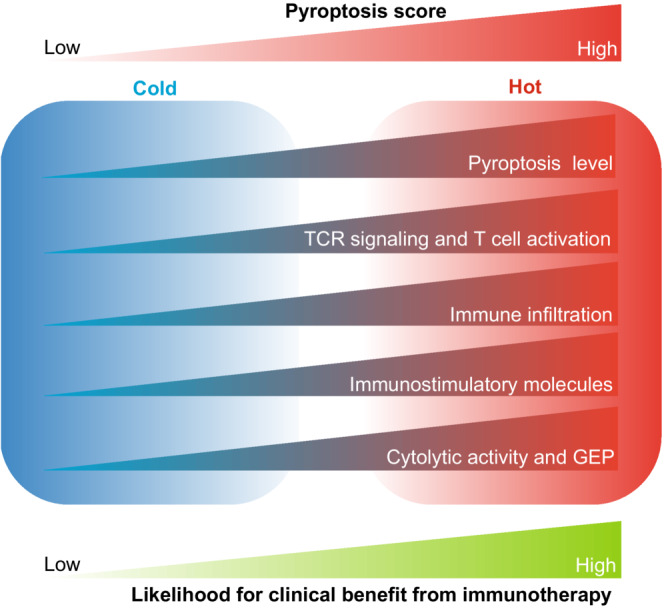
Summary graphic showing the role of pyroptosis score in immune phenotype and tumor immunotherapy

In summary, these findings support that tumors with high pyroptosis scores tended to develop a “hot” immune phenotype, whereas those with low pyroptosis scores showed a “cold” immune phenotype. Therefore, there was an increased likelihood of clinical benefits of immunotherapy for melanoma patients with high pyroptosis scores (Figure 7).

## 4 DISCUSSION

Pyroptosis has a dual role in tumorigenesis and antitumor immunity across diverse cancers. On the one hand, the release of pyroptosis‐producing cytokines could drive the accumulation of immunosuppressive cells (such as MDSC, T_reg_, and M2 cells) and regulate the crosstalk between tumor cells and TME components, eventually leading to tumorigenesis and progression.^14^ On the other hand, pyroptosis could enhance the antitumor effect via recruiting adaptive immune cells, activating cytolytic lymphocytes, and promoting the phagocytosis of macrophages.^16^ Despite these advances, most studies focus on a single infiltrating immune cell or pyroptosis‐related gene. Therefore, an integrated analysis for investigating the role of pyroptosis in TME in melanoma is urgently needed. Furthermore, it remains unclear whether pyroptosis‐related genes could be applied in clinical practice to predict clinical outcomes from immunotherapy. Therefore, constructing a pyroptosis‐based model for immunotherapy will deepen our understanding of the clinical application of pyroptosis in tumor immunotherapy and assist oncologists in identifying the advantage population with clinical benefits from immunotherapy.

In this study, we identified a predictive model using four pyroptosis‐related genes for melanoma patients with immunotherapy. The predictive efficiency of the model for clinical benefit and survival outcomes from immunotherapy was explored and validated in four independent cohorts. Although the immunostimulatory role of pyroptosis has been revealed in melanoma,^38^, ^39^ this study highlights the clinical application of the pyroptosis‐based model in tumor immunotherapy. We observed that the pyroptosis‐based model achieved an effective and robust prediction for clinical benefit, OS, and PFS in the Gide, Lauss, Liu, Nathanson, and meta cohorts. To our knowledge, it is the first time to investigate the predictive value of the pyroptosis‐based model for immunotherapy efficacy in melanoma based on multiple immunotherapy cohorts. Furthermore, it is worth noting that the pyroptosis‐based model only depends on the gene panel's expression level, which is low‐cost and convenient in clinical application. Besides, we also evaluated the association between the pyroptosis‐based model and survival outcomes for melanoma patients without immunotherapy using the TCGA‐SKCM cohort. Interestingly, the prognostic value of the model was not significant (Figure S3), suggesting that the pyroptosis‐based model was a predictive biomarker for immunotherapy instead of a prognostic biomarker in melanoma. Therefore, on the other hand, it is recommended for patients with high pyroptosis scores rather than those with low scores to receive immunotherapy in clinical practice; on the other hand, increasing the pyroptosis level might be a promising strategy to improve sensitivity to immunotherapy for melanoma patients. Converting tumor immunophenotype from “cold” to “hot” by triggering pyroptosis may be a potentially effective strategy for tumors with immune‐desert characteristics. Recent studies have developed novel concepts and methods to integrate pyroptosis‐based therapy with immunotherapy to cure tumors. Zhao et al. designed a pyroptosis‐associated biomaterial nanoparticle to improve the immunotherapy efficacy for tumors with low immunogenicity.^15^ Zhang et al. developed a covalent organic framework for inducing pyroptosis and triggering durable antitumor immunity to boost tumor immunotherapy.^40^ Yang et al. summarized a series of compounds that could activate pyroptosis in cancers,^41^ combined with immunotherapy to exert an antitumor effect. Therefore, combining pyroptosis‐inducing therapy with immunotherapy might be a promising strategy for patients with low pyroptosis scores.

The development of the pyroptosis‐based model has become a hot spot in melanoma research. Cao et al. constructed a prognostic model of genes associated with pyroptosis in melanoma.^19^ Similarly, Zhang et al. also developed a pyroptosis‐related gene signature to predict the prognosis of melanoma patients.^38^ Wang et al. developed a pyroptosis model for predicting the characteristics of the immune microenvironment, the sensitivity to immunotherapy, and the prognosis of melanoma patients.^42^ Meng et al. characterized the pyroptosis‐related immune infiltration landscape in melanoma.^39^ Different from Zhang et al.’s and Wang et al.’s models, our pyroptosis‐based model focused on the predictive efficacy of clinical benefit from immunotherapy in melanoma rather than the prognosis of melanoma patients. More importantly, previous models were constructed based on the clinical cohorts where most patients did not receive immunotherapy, such as TCGA or GEO datasets. On the contrary, our study developed and validated the pyroptosis‐based model based on the immunotherapy cohorts. It is the first time to systematically evaluate the predictive accuracy of the pyroptosis‐based model using four independent immunotherapy cohorts. Summarily, we considered that the predictive value of our model was more reliable and robust than previous models.

Previous studies have revealed a complex molecular mechanism underlying the antitumor effect of pyroptosis. First, the production of proinflammatory cytokines and immunostimulatory molecules, such as IL‐1β,^43^ IL‐18,^44^ HMGB1,^45^ and ATP,^46^ could activate cytotoxic CD8^+^ T cells and Th1 CD4^+^ T cells but inhibit the differentiation of regulatory T cells, eventually leading to the antitumor immune response. Second, GSDM protein could activate the tumor‐infiltrating lymphocytes in TME and upregulate the expression of immune effectors, including IFN‐γ, GZMB, TNF‐α, and PRF.^47^, ^48^ Besides, pyroptotic macrophages could trigger the cytolytic activity of NK cells against cancer cells.^49^ These findings indicated that tumors with high pyroptosis levels harbored a “hot” immunophenotype, with great potential for immunotherapy response. We also explored the association between the pyroptosis‐based model and various aspects of immunological characteristics in TME to explore the molecular mechanism underlying the predictive value for immunotherapy. It was found that a number of immune‐related signaling pathways were significantly enriched in tumors with high pyroptosis scores, such as antigen processing cross presentation, TCR signaling, and cytokine signaling in immune system. Furthermore, tumors with high pyroptosis scores harbored more abundant infiltration levels of cytotoxic CD8^+^ T cells and activated CD4^+^ T cells. In contrast, tumors with low pyroptosis scores contained more resting immune cells, such as naïve CD4^+^ T cells and macrophage M0. At the level of molecules, we observed positive correlations between pyroptosis score and various immune effectors, including activated DC cell markers, antigen‐processing related genes, CD8^+^ T cell effectors, genes downstream of IFN‐γ, NK cell markers, and immune checkpoints. It could be explained that tumors with high pyroptosis scores, featured by the immune‐inflamed phenotype, harbored a higher likelihood of clinical benefit from immunotherapy.

Four pyroptosis‐related genes were enrolled in the predictive model, including CASP5, NLRP6, NLRP7, and PYCARD. The biological functions of these genes have been reported in previous studies. CASP5, encoding a member of the caspase family, plays a key mediator in programmed cell death.^50^ CASP5 initiates the cleavage of GSDMD and the production of the N‐terminal GSDMD moiety that binds to membranes and forms pores, eventually leading to cell expansion and rupture.^51^ Hu et al. found that patients with high expression of CASP5 had a worse survival outcome than those with low expression in kidney carcinoma.^52^ Ulybina et al. found that coding polymorphisms in CASP5 might be a key regulator in predisposition to lung cancer.^53^ NLRP6 is known as a sensor element in the NLRP6 inflammasome.^54^ It promotes the polymerization of the inflammasome to participate in innate immunity and inflammation.^55^ Patients with low expression of NLRP6 showed a worse OS in multiple cancer types, such as liver cancer,^56^ breast cancer,^57^ and gastric cancer.^58^ NLRP7, another member of the NLR family, is involved in activating proinflammatory caspases.^59^ Li et al. demonstrated that NLRP7 promotes CRC progression by polarizing macrophages M2.^60^ Reynaud et al. found that NLRP7 promoted tumorigenesis and progression of choriocarcinoma by constructing an immunosuppressive TME.^61^ Interestingly, in this study, NLRP7 took the largest coefficient in the pyroptosis‐based model, which was positively correlated with an immune‐inflamed TME in melanoma. PYCARD, encoding an adaptor protein, functions as a key regulator in inflammation and programmed cell death.^62^ A prior study showed that elevated expression of PYCARD contributed to the tumor cell growth and poor prognosis of pancreatic cancer.^63^ Furthermore, Strickler et al. identified the diagnostic value of PYCARD in melanoma using immunohistochemical analysis.^64^ Nevertheless, the biological functions of these pyroptosis‐based genes involved in tumor immunity and immunotherapy have rarely been investigated in melanoma, deserving further investigation in vitro/in vivo experiments.

This study had several limitations. Firstly, the best cutoff value of the pyroptosis score remains a mystery for clinical application of the pyroptosis‐based model. Secondly, there are several potential confounding factors, including the heterogeneity of different studies, the selection bias of cohorts, and the batch effect of RNA‐seq data from different platforms. Thirdly, although our research has preliminarily established the association between the model and immunotherapy, it remains unclear which immunotherapy strategies are best suited for this model. In addition, it is valuable to explore the molecular mechanism underly the association between these pyroptosis‐related genes and tumor immunotherapy in the future. Besides, although the prediction capability of the pyroptosis‐based model has been explored in four independent immunotherapy cohorts, it would be ideal if our findings could be validated in prospective, large‐scale, and multi‐center studies. Furthermore, limited by the incomplete clinical information, the pyroptosis‐based model established in this study was only based on gene expression. It would be better to develop a predicting model based on gene and clinical data, which contained more comprehensive features associated with immunotherapy. In the future, a combined model might better assist oncologists in identifying the advantage population with clinical benefits from immunotherapy.

In conclusion, our study developed a reliable and robust model based on four pyroptosis‐related genes to predict clinical outcomes from immunotherapy in melanoma. Comprehensive analysis of four independent cohorts consistently supported that patients with high pyroptosis scores had favorable clinical outcomes from immunotherapy than those with low pyroptosis scores. Furthermore, we comprehensively analyzed the TME characteristics associated with pyroptosis score to explain the predictive and prognostic value of the model for immunotherapy. These findings may assist oncologists in predicting immunotherapeutic efficacy and stratifying melanoma patients, which deserve further investigations in the future.

## AUTHOR CONTRIBUTIONS

Haiyong Wang designed the study. Guanghao Wu, Biying Chen, and Junjie Jiang analyzed the data and wrote the manuscript. Guanghao Wu, Junjie Jiang, Biying Chen, and Yiran Chen drew figures. Guanghao Wu, Biying Chen, and Yanyan performed a literature search. Junjie Jiang, Yanyan Chen, and Haiyong Wang reviewed the manuscript. All authors have read and approved the final version of the manuscript.

## FUNDING INFORMATION

This research was funded by the Key Research and Development Program of Science and Technology Department of Zhejiang Province (2018C03022).

## CONFLICT OF INTEREST

The authors declare that they have no competing interests.

## ETHICS STATEMENT

The authors declare human ethics approval was not needed for this study.

## Supporting information


Figures S1‐S4

Tables S1‐S2

## Data Availability

The RNA‐seq data with annotated clinical information in three immunotherapy cohorts (the Gide, Liu, and Nathanson cohorts) were obtained from the linked supplemental tables in the original references. The data of the Lauss cohort were downloaded from the GEO database (GSE100797). The data of the TCGA‐SKCM cohort was downloaded from the cBioportal database (https://www.cbioportal.org/).
