# Supplementary material for: Identification of a pyroptosis‐based model for predicting clinical outcomes from immunotherapy in patients with metastatic melanoma
Source: Cancer Med. 2022 Sep 23;12(4):4921–37. doi: 10.1002/cam4.5178 (PMC9972144; doi:10.1002/cam4.5178)
Supplement: Supplementary file 1 — Figures S1‐S4 [file CAM4-12-4921-s001.pdf]

Supplementary Figure

Figure S1. ROC curve showing the prediction of durable clinical benefit by pyroptosis score in the combined cohort.

Figure S2. ROC curve showing the prediction of durable clinical benefit by four machine learning models in the Gide, Lauss, Liu, and Nathanson cohorts. (A) RF; **(B)** SVM; **(C)** ANN; **(D)** KNN. RF, random forests; SVM, supporting vector machines; ANN, artificial neural networks; KNN, K-nearest neighbor.

Figure S3. The association of pyroptosis score with OS (A) and PFS (B) in TCGA-SKCM. CI, confidence interval; HR, hazard ratio; OS, overall survival; PFS, progression-free survival.

Figure S4. The predictive and prognostic value of pyroptosis score in patients with gastric cancer or clear cell renal cell carcinoma receiving immunotherapy. (A-B) ROC curve showing the prediction of durable clinical benefit by pyroptosis score in the Kim **(A)** and Braun **(B)** cohorts. **(C-D)** Kaplan-Meier analysis showing the association of pyroptosis score with OS **(C)** and PFS **(D)** in the Braun cohort. The data of survival outcomes were not provided from the Kim cohort. CI, confidence interval; HR, hazard ratio; OS, overall survival; PFS, progression-free survival.

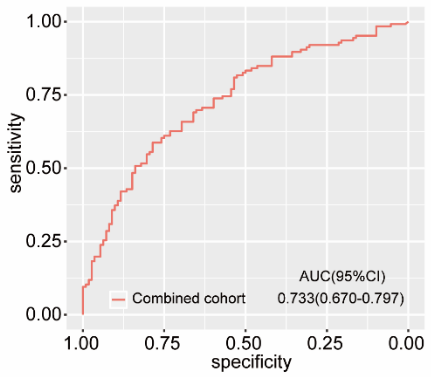

Figure S1. ROC curve showing the prediction of durable clinical benefit by pyroptosis score in the combined cohort.

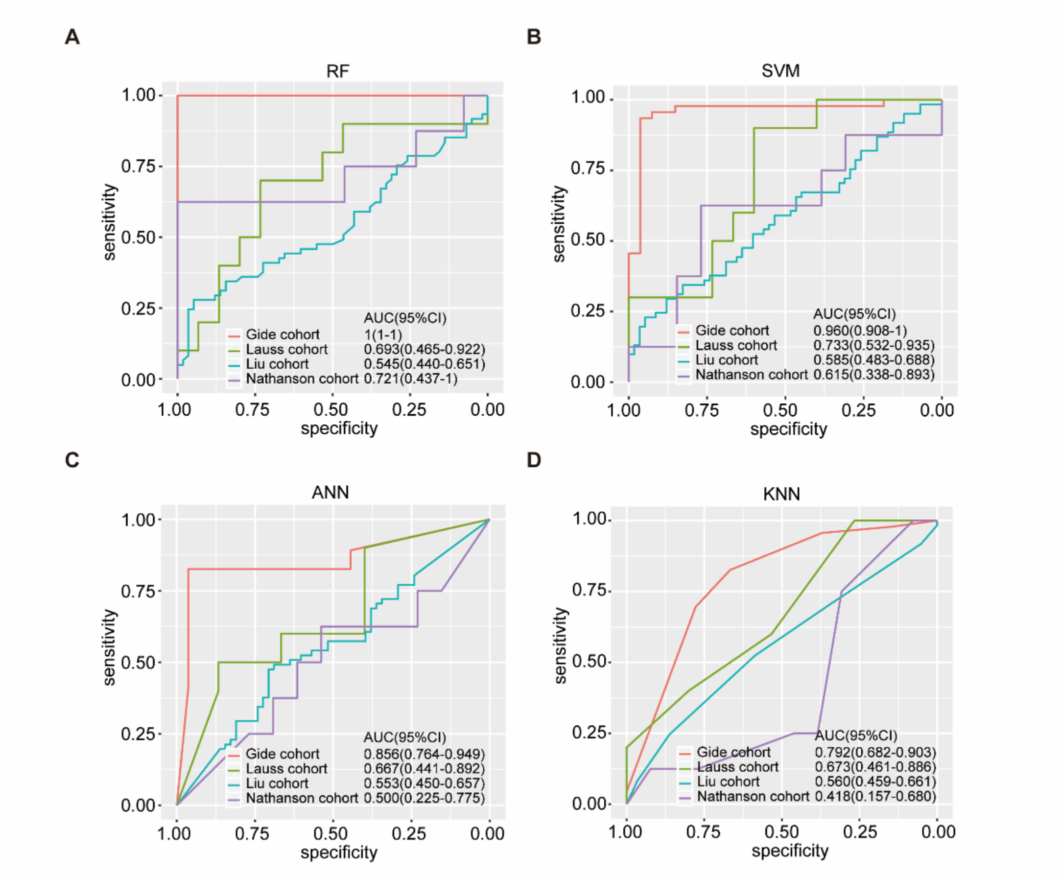

Figure S2. ROC curve showing the prediction of durable clinical benefit by four machine learning models in the Gide, Lauss, Liu, and Nathanson cohorts. (A) RF; **(B)** SVM; **(C)** ANN; **(D)** KNN. RF, random forests; SVM, supporting vector machines; ANN, artificial neural networks; KNN, K-nearest neighbor.

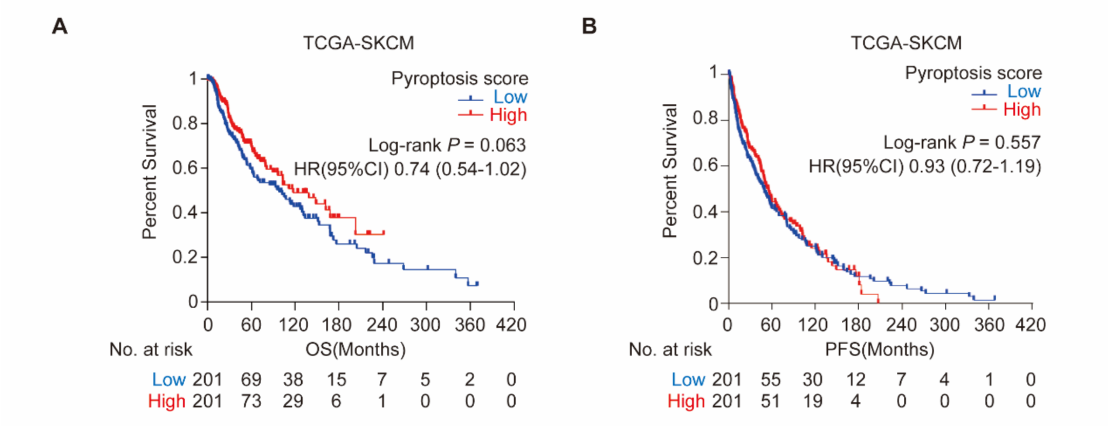

Figure S3. The association of pyroptosis score with OS (A) and PFS (B) in TCGA-SKCM. CI, confidence interval; HR, hazard ratio; OS, overall survival; PFS, progression-free survival.

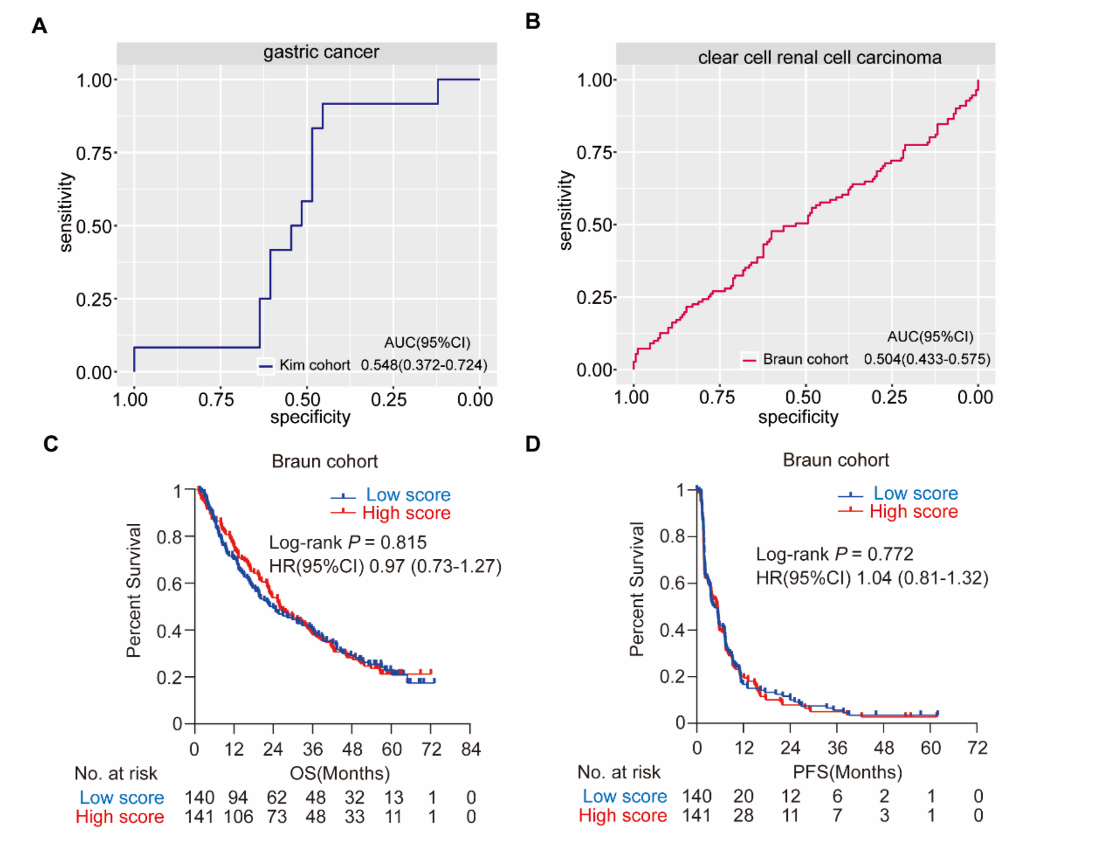

Figure S4. The predictive and prognostic value of pyroptosis score in patients with gastric cancer or clear cell renal cell carcinoma receiving immunotherapy. **(A-B)** ROC curve showing the prediction of durable clinical benefit by pyroptosis score in the Kim **(A)** and Braun **(B)** cohorts. **(C-D)** Kaplan-Meier analysis showing the association of pyroptosis score with OS **(C)** and PFS **(D)** in the Braun cohort. The data of survival outcomes were not provided in the Kim cohort. CI, confidence interval; HR, hazard ratio; OS, overall survival; PFS, progression-free survival.
