# Supplementary material for: Identification of a pyroptosis‐based model for predicting clinical outcomes from immunotherapy in patients with metastatic melanoma
Source: Cancer Med. 2022 Sep 23;12(4):4921–37. doi: 10.1002/cam4.5178 (PMC9972144; doi:10.1002/cam4.5178)
Supplement: Supplementary file 2 — Tables S1‐S2 [file CAM4-12-4921-s002.docx]

| **Table S1. The 33 pyroptosis-related genes used in this study.** | | |  |
| --- | --- | --- | --- |
| **Gene symbol** | **Entrez Gene ID** | **Description** | |
| *AIM2* | 9447 | Absent In Melanoma 2 | |
| *CASP1* | 834 | Caspase 1 | |
| *CASP3* | 836 | Caspase 3 | |
| *CASP4* | 837 | Caspase 4 | |
| *CASP5* | 838 | Caspase 5 | |
| *CASP6* | 839 | Caspase 6 | |
| *CASP8* | 841 | Caspase 8 | |
| *CASP9* | 842 | Caspase 9 | |
| *ELANE* | 1991 | Elastase, Neutrophil Expressed | |
| *GPX4* | 2879 | Glutathione Peroxidase 4 | |
| *GSDMA* | 284110 | Gasdermin A | |
| *GSDMB* | 55876 | Gasdermin B | |
| *GSDMC* | 56169 | Gasdermin C | |
| *GSDMD* | 79792 | Gasdermin D | |
| *GSDME* | 1687 | Gasdermin E | |
| *IL18* | 3606 | Interleukin 18 | |
| *IL1B* | 3553 | Interleukin 1 Beta | |
| *IL6* | 3569 | Interleukin 6 | |
| *NLRC4* | 58484 | NLR Family CARD Domain Containing 4 | |
| *NLRP1* | 22861 | NLR Family Pyrin Domain Containing 1 | |
| *NLRP2* | 55655 | NLR Family Pyrin Domain Containing 2 | |
| *NLRP3* | 114548 | NLR Family Pyrin Domain Containing 3 | |
| *NLRP6* | 171389 | NLR Family Pyrin Domain Containing 6 | |
| *NLRP7* | 199713 | NLR Family Pyrin Domain Containing 7 | |
| *NOD1* | 10392 | Nucleotide Binding Oligomerization Domain Containing 1 | |
| *NOD2* | 64127 | Nucleotide Binding Oligomerization Domain Containing 2 | |
| *PJVK* | 494513 | Pejvakin | |
| *PLCG1* | 5335 | Phospholipase C Gamma 1 | |
| *PRKACA* | 5566 | Protein Kinase CAMP-Activated Catalytic Subunit Alpha | |
| *PYCARD* | 29108 | PYD And CARD Domain Containing | |
| *SCAF11* | 9169 | SR-Related CTD Associated Factor 11 | |
| *TIRAP* | 114609 | TIR Domain Containing Adaptor Protein | |
| *TNF* | 7124 | Tumor Necrosis Factor | |

| **Table S2. Correlation analysis between pyroptosis score and immune effectors** | | | |
| --- | --- | --- | --- |
| **Category** | **Variable** | **Spearman’s R** | ***P*** |
| Activated DC marker | *BATF3* | 0.317088 | 5.87E-07 |
|  | *IRF8* | 0.641157 | 5.91E-29 |
|  | *THBD* | 0.218437 | 0.000691 |
|  | *CLEC9A* | 0.482035 | 2.98E-15 |
|  | *XCR1* | 0.557342 | 7.99E-21 |
| Antigen processing machinery | *B2M* | 0.483597 | 2.36E-15 |
|  | *HLA-A* | 0.438331 | 1.35E-12 |
|  | *HLA-B* | 0.522072 | 4.84E-18 |
|  | *HLA-C* | 0.495373 | 3.85E-16 |
|  | *TAP1* | 0.610779 | 9.99E-26 |
|  | *TAP2* | 0.489136 | 1.01E-15 |
|  | *TAPBP* | 0.52442 | 3.23E-18 |
|  | *HLA-DPA1* | 0.578331 | 1.21E-22 |
|  | *HLA-DPB1* | 0.561688 | 3.44E-21 |
|  | *HLA-DQA2* | 0.414054 | 2.83E-11 |
|  | *HLA-DQB1* | 0.355305 | 1.72E-08 |
|  | *HLA-F* | 0.560748 | 4.13E-21 |
|  | *HLA-G* | 0.220131 | 0.000626 |
|  | *HLA-DMA* | 0.538799 | 2.55E-19 |
|  | *HLA-DMB* | 0.542361 | 1.33E-19 |
|  | *HLA-DOA* | 0.559458 | 5.31E-21 |
|  | *HLA-DOB* | 0.630298 | 9.26E-28 |
| CD8^+^ T effector | *CD8A* | 0.649199 | 7.17E-30 |
|  | *CD8B* | 0.626934 | 2.13E-27 |
|  | *CD3D* | 0.656319 | 1.05E-30 |
|  | *CD3E* | 0.67191 | 1.29E-32 |
|  | *CD2* | 0.696638 | 6.81E-36 |
|  | *CD27* | 0.667928 | 4.08E-32 |
| IFN-gamma signature | *IDO1* | 0.604888 | 3.85E-25 |
|  | *CXCL10* | 0.577842 | 1.34E-22 |
|  | *CXCL9* | 0.592927 | 5.48E-24 |
|  | *HLA-DRA* | 0.563932 | 2.21E-21 |
|  | *IFNG* | 0.556196 | 9.95E-21 |
|  | *CMKLR1* | 0.518071 | 9.55E-18 |
|  | *PSMB10* | 0.576084 | 1.92E-22 |
|  | *STAT1* | 0.538271 | 2.80E-19 |
|  | *HLA-DQA1* | 0.413835 | 2.91E-11 |
|  | *HLA-DRB1* | 0.533067 | 7.11E-19 |
| NK cells marker | *NKG7* | 0.592716 | 5.74E-24 |
|  | *HLA-E* | 0.521388 | 5.44E-18 |
| Cytolytic activity | *GZMA* | 0.645529 | 1.89E-29 |
|  | *GZMB* | 0.637281 | 1.60E-28 |
|  | *GZMK* | 0.602533 | 6.56E-25 |
|  | *PRF1* | 0.623099 | 5.41E-27 |
| Immune checkpoint | *CD274* | 0.590121 | 1.01E-23 |
|  | *PDCD1* | 0.592214 | 6.40E-24 |
|  | *PDCD1LG2* | 0.562146 | 3.14E-21 |
|  | *LAG3* | 0.596325 | 2.61E-24 |
|  | *CTLA4* | 0.465126 | 3.53E-14 |
